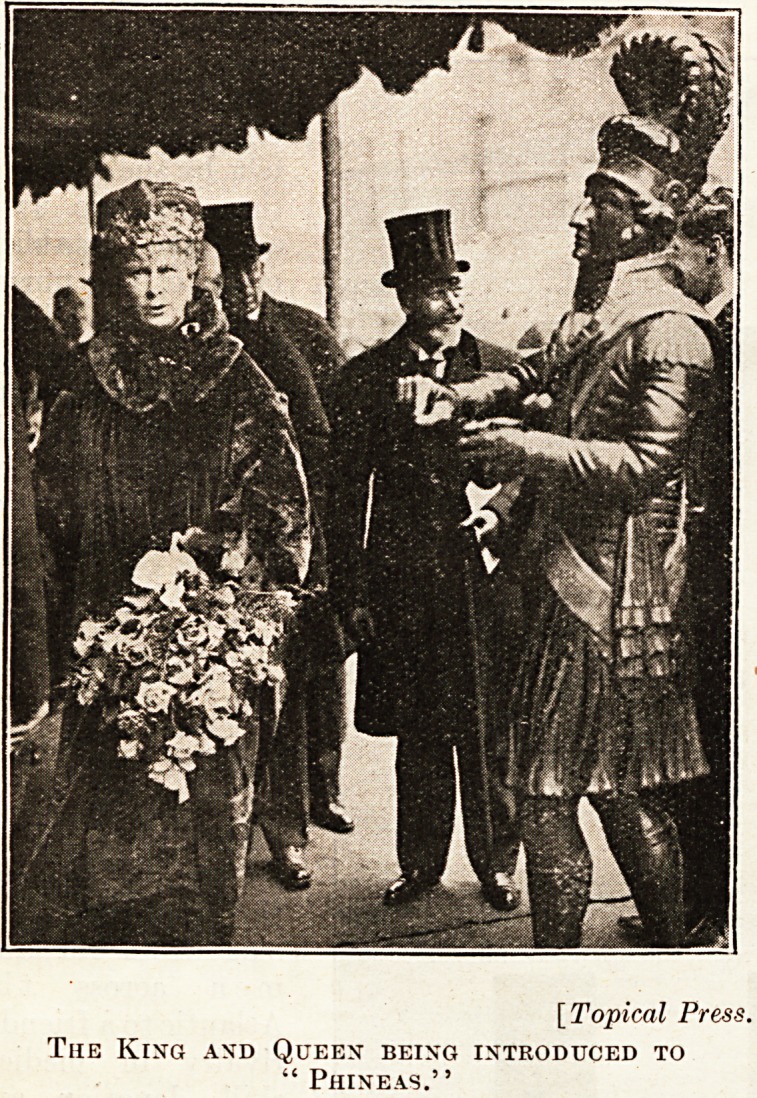# Extension of University College Hospital

**Published:** 1923-07

**Authors:** 


### EXTENSION OF UNIVERSITY COLLEGE HOSPITAL.

### THE KING AND THE ROCKEFELLER GIFT.

IN the presence of a distinguished gathering and amid much dignified ceremonial the King and Queen laid the foundation stones of the new obstetric hospital and the new nurses' home of University College Hospital on May 31. This, as the King in his most impressive address pointed out, "is no ordinary extension of a hospital or college. The vast scale of the new development which we are inaugurating would be enough in itself to render it remarkable. There can be but few instances on record in which any foundation has received, like this College and Medical School, £1,200,000 from a single benefactor in a single gift. (Cheers.) And the magnificent generosity of the Rockefeller Trustees is the more impressive since it is bestowed by a citizen of the United States of America upon a college and hospital in London, and thus upon the people of Great Britain and the Empire."

THE NEW ANATOMY BUILDING.

The new buildings will face each other in Huntley Street, Gower Street, and a pavilion, the shape of a Maltese Cross, was erected to cover them both so that the proceedings really took place in the middle of the street. The pavilion with its tiers of seats rising to the roof was bright with the scarlet of gowns and hoods, and the nurses of the hospital were squeezed into every available corner, while some of them formed a Guard of Honour. Loud cheers from Tottenham Court Road heralded the approach of the King and Queen, and a great reception awaited them in the marquee, where they took their seats in two large armchairs in the centre of the building. After a welcome by the Duke of Bedford, President of the College, Sir Ernest Hatch, Chairman of the General Committee, read an address to their Majesties to which the King replied.

"I understand," he said, "that this College and Medical School were selected by the Rockefeller Trustees for their benefaction, from among many equally distinguished institutions, partly because the situation is central and yet affords room for expansion, and partly because the close connection of the Hospital and Medical School with the College provides favourable opportunities for that intercourse between medicine and other branches of learning which is the surest defence against the evils of a narrow specialism." His Majesty went on to refer to the interest felt by the Queen in the obstetric unit and the establishment of the nurses' home. He added: "The privilege of accepting this munificent gift of the Rockefeller Trustees imposes obligation upon the staff to fulfil the ideals which it represents, and upon the public to furnish the necessary support entailed. It is inconceivable that Englishmen should decline to welcome this generous challenge from our kinsmen across the Atlantic to a friendly rivalry in medical skill, devotion, and beneficence. I cordially wish God-speed to this great enterprise."

The Bishop of London having offered prayer, the surpliced choir of St. Pancras Church sang the hymn "Praise My Soul the King of Heaven," accompanied by the famous band of the Grenadier Guards. Then came the laying of the foundation stone, which was done in a thoroughly business-like way, the King laying the stone of the obstetric hospital and the Queen the stone of the nurses' home. Within the stone was deposited a copy of the authorised programme of the day, a copy of *The Times* of the day, specimens of the current coins of the realm, and the current edition of the Hospital Report, with list of the staff, &c. The King, by pressing an electric button, next opened the doors of the new anatomy building. The King and Queen, sheltered by a canopy held by students, then visited the new lecture theatre, library and dissecting-room. In the X-rays room they were shown an X-ray photograph of the mummy of Thotmes IV., taken twenty years ago. They also made acquaintance with "Phineas," the mascot of the University.

The anatomy building, also the gift of the Rockefeller Foundation, has been designed by Professor F. M. Simpson F.R.I.B.A., and faces Gower Street. It is planned on the most modern

[*Topical Press.*

THE KING AND QUEEN BEING INTRODUCED TO "PHINEAS."

lines and possesses a particularly fine dissecting-room, with X-ray room adjoining, so that the student, while making a dissection of a particular limb, will be able to study the living model. At the hospital itself, room for an additional 120 beds will be gained by the removal of the nurses' home and residents' quarters, now occupying the south-west wing, and their conversion into wards. A word of praise must be added for the admirable ceremonial and other arrangements made by the hospital authorities.

**Reception at University College.**

The Chairman (Viscount Chelmsford) and members of the University College Committee, the Provost and members of the academic staff will hold a reception at the College on July 7. The new anatomy building and the extensions of the physiology and engineering departments will be open to inspection. Old students of the College who have not recently communicated their addresses are invited to send them, stating year of entry and Faculty, to the Secretary.

## Figures and Tables

**Figure f1:**
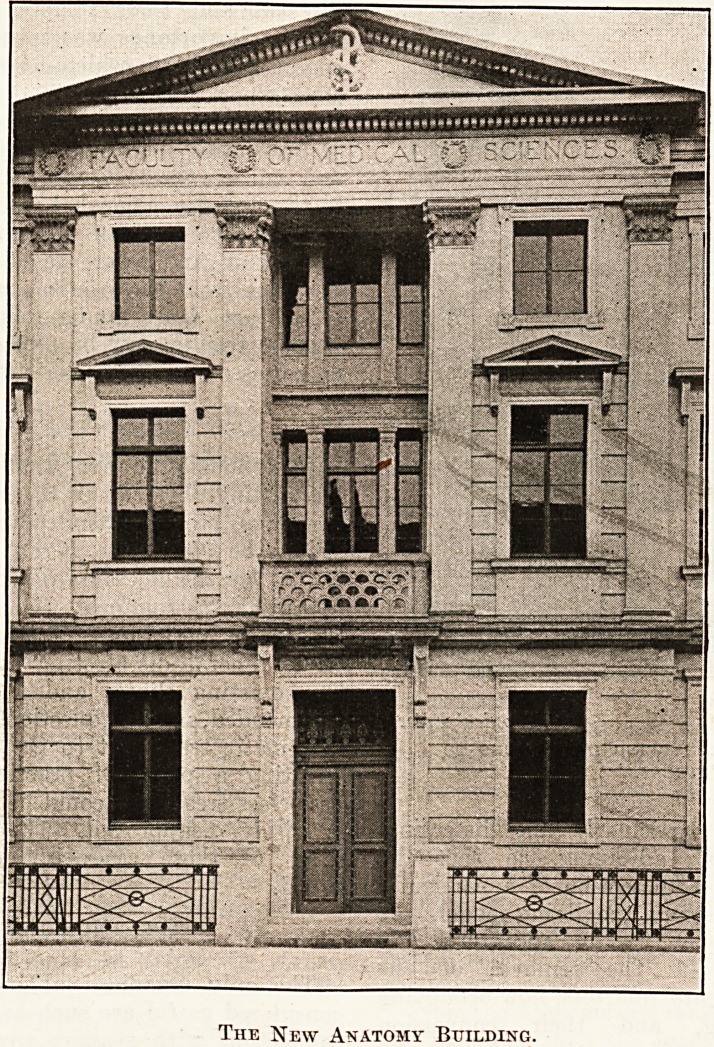


**Figure f2:**